# Barriers and facilitators for medical oncologists in the further implementation of mainstream genetic testing in breast cancer care in the Netherlands

**DOI:** 10.1007/s10689-025-00500-9

**Published:** 2025-10-14

**Authors:** Chiem L de Jong, Gina Schijven, Ellen G Engelhardt, Agnes Jager, Margreet G. E. M. Ausems

**Affiliations:** 1 Division Laboratories, Pharmacy and Biomedical Genetics, Department of Genetics, University Medical Center Utrecht, Heidelberglaan 100, 3584 CX Utrecht, The Netherlands; 2 Division of Psychosocial Research and Epidemiology, The Netherlands Cancer Institute, Plesmanlaan 121, 1066 CX Amsterdam, The Netherlands; 3 Department of Medical Oncology, Erasmus University MC Cancer Institute, Dr. Molewaterplein 40, PO Box 2040, 3000 CA Rotterdam, The Netherlands

**Keywords:** Breast cancer, Genetic testing, Mainstream genetic testing, Barriers and facilitators, Medical oncologists, Disparities.

## Abstract

Only 67% of eligible patients with breast cancer currently undergo genetic testing. Mainstream genetic testing (i.e. healthcare professionals (HCPs) from outside the genetics field providing genetic counselling), also referred to asmainstreaming, leads to increased uptake of genetic testing. Unlike surgical oncologists, medical oncologists have not yet widely adopted mainstreaming. This study aims to identify barriers and facilitators for HCPs, in particular medical oncologists, in advancing the implementation of mainstreaming. Structured interviews were conducted with HCPs (predominantly medical oncologists) using the Constructive Technology Assessment (CTA) framework. Participants were recruited from all regions of the Netherlands, ensuring representation from academic medical centres, teaching hospitals and general hospitals. The interview data was analysed using thematic analysis. In this qualitative study, 12 medical oncologists, three nurse practitioners, three clinical geneticists, two clinical laboratory geneticists and one representative of a health insurer were interviewed. Lack of time and limited knowledge were the most commonly mentioned barriers, whilst effective cross-departmental collaboration and education were the most important facilitators. For medical oncologists specifically, financial compensation for the increased workload and the increasing importance of genetic testing for guiding therapy choices were found to be encouraging factors. This study identified several barriers and facilitators to mainstreaming being implemented by medical oncologists. Education to strengthen oncologists’ skills in discussing germline genetic testing may address knowledge gaps and time barriers. Alongside cross-departmental collaboration, these strategies could streamline genetic testing pathways. Further research should evaluate their implementation and effects on patient experience and equitable access.

**Supplementary Information:**

The online version contains supplementary material available at 10.1007/s10689-025-00500-9.

## Introduction

Germline genetic testing in breast cancer patients provides insights into their risk of developing secondary breast, ovarian or other malignancies, determines surgical and oncological treatment options, and identifies potential cancer risks for family members [1, 2]. However, only 67% of eligible patients currently undergo genetic testing [3, 4]. The aforementioned limited uptake of genetic testing and the limited capacity of genetics departments has created a need for a restructured approach in the organisation of genetic care. Mainstream genetic testing, or mainstreaming, in which genetic testing is discussed and initiated by a non-genetic healthcare professional (HCP), was first introduced for ovarian cancer patients in 2016 [5]. In subsequent years, this approach has been increasingly adopted, e.g. by extending its application to the care of breast cancer patients [6–9].

In the Netherlands, patients with breast cancer can either be referred for genetic testing to a genetics HCP at one of the eight genetics departments or to a genetics HCP at a satellite outpatient clinic in a non-academic hospital. In approximately one-third of Dutch hospitals, genetic testing is conducted through the mainstreaming pathway, most commonly involving surgeons and nurse practitioners from surgical departments [4, 10]. Medical oncologists, however, have not yet widely adopted the mainstreaming pathway. They could however play a crucial role in improving genetic testing uptake, as testing rates among patients with *de novo* metastatic breast cancer (stage IV) remain relatively low compared to those with stage I–III disease [4]. Firstly, patients with stage IV breast cancer often do not visit a surgical oncologist but go straight to the medical oncologist. Also, patients who primarily visited the surgical oncologist but have unexpectedly not undergone genetic testing can be identified by the medical oncologist. Additionally, genetic testing is becoming increasingly important for treatment selection [11, 12], encouraging medical oncologists to discuss and offer genetic testing to their patients. In the Netherlands, we previously explored experiences among surgical oncologists and nurses in breast cancer care who were involved in mainstreaming, which led to positive attitudes towards mainstreaming [10]. However, little is known about barriers and facilitators for further implementation of mainstreaming among HCPs, in particular medical oncologists [13].

This study aims to identify the barriers and facilitators faced by non-genetic HCPs (e.g. medical oncologists and nurse practitioners from oncology departments) and other stakeholders (e.g. clinical geneticists, clinical laboratory geneticists and health insurers) in further implementing mainstreaming in breast cancer care. Once these barriers and facilitators are known, opportunities can be explored to enhance the motivation to start mainstreaming among non-genetic HCPs, particularly those in oncology.

## Methods

### Study design and interview guide

The Constructive Technology Assessment (CTA) framework was used for structuring the interviews [14]. This framework focuses on organisational, clinical, economic and patient-related aspects [15]. These aspects, combined with demographical questions about the participant, form the foundation of the interview guide (Appendix 1). These five areas were complemented with additional in-depth questions to encourage further discussion. The in-depth questions in the interview guide were based on the results of a questionnaire that was sent to HCPs by the National Breast Cancer Consultation Netherlands (NABON) in 2022. This questionnaire included questions on HCPs’ experiences with and opinions on mainstreaming of genetic care.

### Study population

We invited HCPs from a range of disciplines, both directly and indirectly involved in breast cancer care, to participate in interviews. Participants were selected through purposive sampling via the national mainstreaming working group from the Dutch Clinical Genetics Society. Medical oncologists and/or nurse practitioners were selected from two hospitals (one from an academic or teaching hospital and one from a general hospital) in each of the regions associated with the eight genetics departments in the Netherlands. Other stakeholders who were interviewed included clinical geneticists, clinical laboratory geneticists and a representative from a health insurer. We completed the recruitment of stakeholders once we had gathered a representative sample of participants from diverse disciplines, hospitals and regions across the Netherlands, ensuring sufficient variation to reach theoretical saturation.

### Data analysis

All interviews were transcribed verbatim (CJ). A coding tree was created in the data analysis. Data was analysed using Nvivo version 14 according to systematic thematic analysis as described by Braun and Clarke [16]. The following steps were followed: (1) familiarisation with the data; (2) generating initial codes; (3) searching for themes; (4) reviewing themes; (5) defining and naming themes; (6) producing the report. The first six interviews were doubly coded by two researchers (CJ and GS), after which inconsistencies were discussed. The final 15 interviews were coded independently by one researcher (either CJ or GS), with any uncertainties or ambiguous items being collaboratively discussed to reach consensus. One researcher (GS) had prior experience in qualitative data analysis, while the other (CJ) was new to this method. To ensure rigour, coding decisions and interpretations were regularly reviewed and discussed.

## Results

### Characteristics, participants and current practice

We conducted in-depth, semi-structured interviews with 21 stakeholders between February 2024 and July 2024 (Table 1). The participants were 12 medical oncologists, three nurse practitioners, three clinical geneticists, two clinical laboratory geneticists and one health insurer. All healthcare providers were employed in the Netherlands by either an academic hospital, a teaching hospital or a general hospital and had varying amounts of experience in breast cancer care. Mainstreaming had been implemented in the hospitals of 13 of the 15 medical oncologists and nurse practitioners (87%). However, only three medical oncologists and one nurse practitioner stated that they were actually involved in discussing and ordering genetic testing. The other nine medical oncologists and nurse practitioners stated that surgical oncologists and nurse practitioners from the surgical department were responsible for discussing and ordering genetic testing. Genetic testing was discussed in a multidisciplinary team in the hospitals of 13 of the 20 participating HCPs (65%). However, only five of these 13 HCPs stated that a clinical geneticist was involved in the multidisciplinary team.

**Table 1:** Demographic overview of participants

Characteristic	Medical oncologists and nurse practitioners (*n* = 15), no. (%)	Clinical geneticists (*n* = 3), no. (%)	Clinical laboratory geneticists (*n* = 2), no. (%)	Health insurer (*n* = 1), no. (%)
*Gender*				
Male	2 (13)	0 (0)	2 (100)	0 (0)
Female	13 (87)	3 (100)	0 (0)	1 (100)
*Work experience in the field*				
< 5 years	3 (20)	0 (0)	0 (0)	0 (0)
5–10 years	2 (13)	2 (67)	1 (50)	0 (0)
10–15 years	3 (20)	0 (0)	1 (50)	1 (100)
> 15 years	7 (47)	1 (33)	0 (0)	0 (0)
*Hospital*				
Academic	4 (27)	3 (100)	2 (100)	–
Teaching	4 (27)	0 (0)	0 (0)	–
General	7 (47)	0 (0)	0 (0)	–

### Themes

**Fig. 1:**
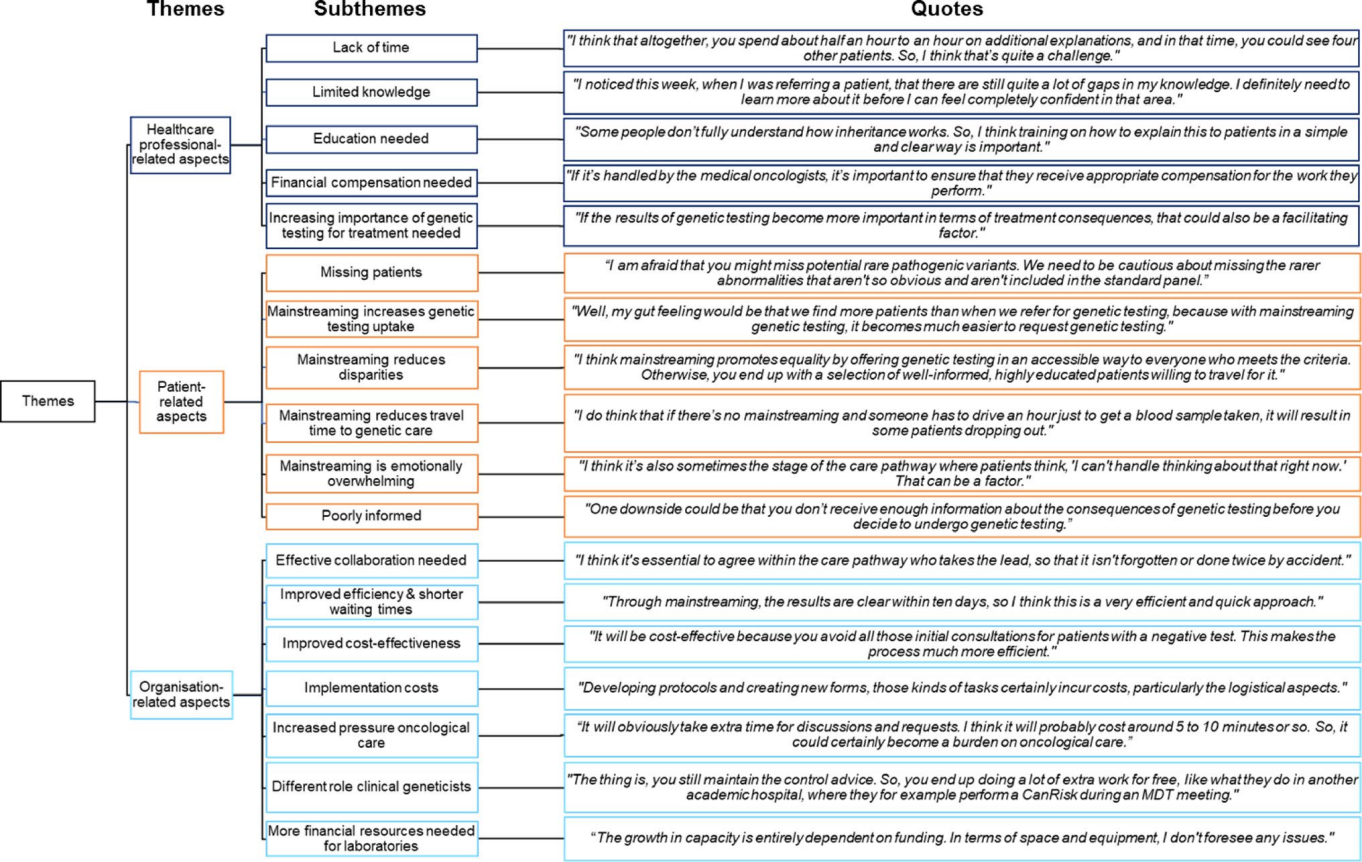
Overarching themes, subthemes and corresponding quotes

We identified three overarching themes and 18 subthemes. In the following section, all subthemes are described according to the CTA framework, with a distinction between ‘healthcare professional-related aspects’, ‘patient-related aspects’ and ‘organisation-related aspects’. The themes identified from the interviews are presented in Fig. 1, each accompanied by a representative quote. These themes are also summarised in Table 2, along with the number of stakeholders who referenced each theme. Direct quotations are referenced by their corresponding numbers in Appendix 2 (e.g. Q1–5), providing the source for each statement.

**Table 2:** Themes identified for further implementation of mainstreaming in breast cancer care

	Themes	Medical oncologists & nurse practitioners (*n* = 15)	Clinical geneticists (*n* = 3)	Clinical laboratory geneticists (*n* = 2)	Health insurer (*n* = 1)
No.	*Theme: Healthcare professional-related aspects*	*N* ^a^	*N*	*N*	*N*
1	Lack of time (barrier)	**10** ^**b**^	**–**	–	**-**
2	Limited knowledge (barrier)	**9**	–	–	–
3	Education needed (facilitator)	**10**	–	–	–
4	Financial compensation needed (facilitator)	4	–	–	–
5	Increasing importance of genetic testing for treatment needed (facilitator)	3	–	–	–
	*Theme: Patient-related aspects*				
6	Missing patients (adverse effect)	1	**2**	0	0
7	Mainstreaming increases genetic testing uptake (benefit)	**11**	**2**	**2**	**1**
8	Mainstreaming is emotionally overwhelming (adverse effect)	7	1	0	0
9	Poorly informed (adverse effect)	**8**	**2**	0	0
10	Mainstreaming reduces disparities (benefit)	**8**	**2**	**2**	**1**
11	Mainstreaming lowers burden by reducing travel time to genetic care (benefit)	6	1	0	0
	*Theme: Organisation-related aspects*				
12	Effective collaboration needed (facilitator)	**8**	**2**	0	**1**
13	Improved efficiency and shorter waiting times (benefit)	7	**3**	**1**	0
14	Improved cost-effectiveness (benefit)	**8**	**2**	0	**1**
15	Implementation costs (adverse effect)	**10**	**3**	**2**	0
16	Increased pressure on oncological care (adverse effect)	6	1	0	0
17	Different role of clinical geneticists	–	1	–	–
18	More financial resources needed for laboratories (facilitator)	–	–	**2**	–

^a^ N = Number of interviews in which this theme was mentioned within a stakeholder group

^b^ All numbers in bold are factors mentioned by ≥ 50% of the respondents in the stakeholder group, and 6/15 < 50%.

### Healthcare professional-related aspects

We identified five subthemes related to the perspective of the HCP, comprising two barriers and three facilitators.

#### Lack of time (barrier)

Lack of time was the most important barrier to further implementation of mainstreaming, mentioned by almost all medical oncologists and nurse practitioners. This could be due to either the additional administrative burden or, more importantly, an increase in consultation time (Appendix 2, Q1-5). A medical oncologist and a nurse practitioner already taking part in mainstreaming stated that the extra time investment was acceptable.

#### Limited knowledge (barrier)

The majority of medical oncologists and nurse practitioners listed limited knowledge of breast cancer genetic counselling and genetic testing as a barrier. Limited ability in understanding the broad gene panels, explaining genetic testing, mapping a complete family history and answering patients’ specific genetics questions were also noted (Q6-9). One medical oncologist involved in mainstreaming noted that, while clinical geneticists possess more specialized expertise in the field, medical oncologists have sufficient knowledge to counsel patients effectively about genetic testing. Another medical oncologist involved in mainstreaming expressed a similar view, describing counselling about genetic testing as an integral component of breast cancer care.

#### Education needed (facilitator)

The majority of participants stated that they felt confident in discussing genetic testing. However, education was highlighted as an important facilitator, noted by a third of medical oncologists and nurse practitioners. Most of them had a positive attitude to education about genetic testing. The key topic to be covered during education was the impact of genetic testing on the patient, including social, practical, financial and emotional consequences (Q10-13). Most of those willing to undergo education about genetic testing felt that a single educational session was sufficient, while others preferred ‘follow-up’ education or ‘learning on the job’ (Q14-15). Others found the availability of an eligibility criteria checklist and clear patient information to be helpful. Those hesitant about participating in the education were unsure of what could be gained from it.

#### Financial compensation needed (facilitator)

Some medical oncologists said that financial compensation would be appropriate to account for the additional workload associated with mainstreaming (Q16-18).

#### Increasing importance of genetic testing for treatment needed (facilitator)

Some medical oncologists also stated that the increasing importance of genetic testing for treatment selection could be seen to be encouraging HCPs to participate in mainstreaming, as in this case genetic testing would become more relevant for medical oncologists (Q19-20).

### Patient-related aspects

For the patient-related aspects, we identified six subthemes, comprising three benefits and three adverse effects.

#### Missing patients (adverse effect)

The majority of participants stated that their perception was that only a limited number of eligible patients are not receiving genetic testing (Q21-23). Those who said otherwise noted that older patients (age > 50), patients with amendments to the family history or those who were unwilling to undergo genetic testing themselves are often the ones not receiving genetic testing (Q24-27). According to medical oncologists, eligible patients with metastatic breast cancer are frequently not offered genetic testing. Additionally, two medical oncologists stated that, in some cases, they chose to postpone discussing genetic testing with the patient (Q28-30). This could be because there was already too much to talk about during the first few consultations and it was then forgotten. Two clinical geneticists expressed concerns about potentially overlooking patients if genetic testing is requested by non-genetic HCPs. They worried that important clues in the patient’s family history or pathogenic variants in genes associated with rare cancer predisposition syndromes could be missed (Q31-32).

#### Mainstreaming increases genetic testing uptake (benefit)

Most participants expected mainstreaming to increase the uptake of genetic testing (Q33-37). The most important reason for this was that mainstreaming makes genetic care more accessible, because patients do not need additional visits to another doctor (the clinical geneticist), who is sometimes even in another hospital (Q38-42). Another reason mentioned was that mainstreamed genetic testing would be forgotten less often. A few participants anticipated that the uptake would remain the same, as testing rates were already considered sufficient.

#### Mainstreaming is emotionally overwhelming (adverse effect)

A possible drawback that was noted is that discussing genetic testing with the medical oncologist could be too emotionally overwhelming for patients (Q43-46), given that they are already receiving a large amount of information.

#### Poorly informed (adverse effect)

Another disadvantage highlighted was that patients might be less well informed if genetic testing is discussed by a non-genetic HCP, leaving them with questions and a wish to consult a clinical geneticist (Q47-49).

#### Mainstreaming reduces disparities (benefit)

More than half of the participants expected low socioeconomic status (SES) to have a negative impact on the uptake of genetic testing. Reasons given were that patients with high SES asked for genetic testing themselves more often and knew more about cancer prevalence (specifically breast cancer) in their families (Q50-53). A language barrier in the case of a migrant background or low literacy (including health literacy) was also listed (Q54). One medical oncologist mentioned a possible bias against discussing genetic testing with patients with low SES (Q55). Those who did not believe that SES was related to the likelihood of receiving a genetic test stated that they had no bias if the testing was clinically relevant and genetic testing was performed regardless of the patient’s SES. However, nearly three quarters of participants expected mainstreaming to have a positive effect on increasing accessibility of genetic care for patients with a low SES, making access to genetic testing more equitable (Q56-59).

#### Mainstreaming reduces travel time to genetic care (benefit)

Opinions were divided about the effect that travel time to genetic care has on the likelihood of receiving a genetic test. Some stated that distance could be a barrier for certain patients, often due to limited options or restricted financial means. Others noted that the introduction of video consultations means that travel time is no longer a barrier (Q60-64). A third of the participants expected that introducing mainstreaming would have a positive effect on the accessibility and equitability of genetic care, due to a decrease in travel time and travel costs (Q65-66).

### Organisation-related aspects

We identified seven subthemes related to organisational factors that were one barrier, two facilitators, two benefits and two adverse effects.

#### Effective collaboration needed (facilitator)

The most important facilitator mentioned by six medical oncologists and two clinical geneticists was setting up effective collaboration through well-defined agreements between different hospital departments and between genetic and non-genetic HCPs about the responsibilities for each part of the care pathway (Q67-70). Clear agreements about who is responsible for taking the patient’s family history and who is responsible for ordering genetic testing were mentioned.

#### Improved efficiency and shorter waiting times (benefit)

Most participants expected mainstreaming to be faster and more efficient compared to referring a patient to a clinical geneticist (Q71-76). More than half of the participants expected that implementing mainstreaming would positively impact the capacity and waiting times for genetic care at the genetics department due to reduced referrals for consultations with clinical geneticists (e.g. only patients with a pathogenic variant or with a positive family history) (Q77-81).

#### Improved cost-effectiveness (benefit)

Most participants anticipated that mainstreaming would be cost-effective, with the majority expecting a reduction in costs due to fewer clinical genetics consultations. Additionally, a reduction in the costs to society through breast cancer prevention in diagnosed patients’ families was also noted (Q82-85). Both of the clinical laboratory geneticists noted that there would be a relative decrease in costs, driven by greater efficiency resulting from higher testing rates. These differences, however, were expected to be limited (Q86). The health insurer representative added that the consultation rates for medical oncologists, surgical oncologists and nurse practitioners are lower than those for clinical geneticists (Q87).

#### Implementation costs (adverse effect)

An adverse effect was noted in terms of the implementation costs of mainstreaming: educational, logistical and personnel expenses were identified as the most significant costs (Q88-89). Other challenges listed included an increase in the number of genetic testing requests for ineligible patients, resulting in higher costs (Q90-91). Some also suggested that mainstreaming could lead to higher genetic testing costs due to an increase in testing rates (Q92).

#### Increased pressure on oncological care (adverse effect)

Another potential adverse effect that was mentioned was the increased pressure on oncological care, which was largely due to extended consultation times for oncologists, leading to delays in their schedules (Q93-96).

#### Different role for clinical geneticists (barrier)

A barrier noted by one clinical geneticist was the concern that implementing mainstreaming could shift their role to a more consultative one. This shift could potentially result in the genetics department failing to meet the agreed number of consultations, which in turn might lead to decreased reimbursement through Diagnosis-Treatment Combinations (DBC), the Dutch variation of the Diagnosis-Related Group (DRG) system (Q97).

#### More financial resources needed for laboratories (facilitator)

Both clinical laboratory geneticists noted that the increased number of genetic tests as a result of mainstreaming being implemented could strain the laboratory’s capacity, with financial resources being the primary concern. The increasing number of genetic tests performed for treatment purposes was mentioned as a factor. However, both clinical laboratory geneticists highlighted the potential for increased testing volume if financial resources could be increased. Additionally, both stated that it does not matter (from the laboratory’s perspective) who orders the genetic test (Q98-100).

### Barriers and facilitators

**Fig. 2:**
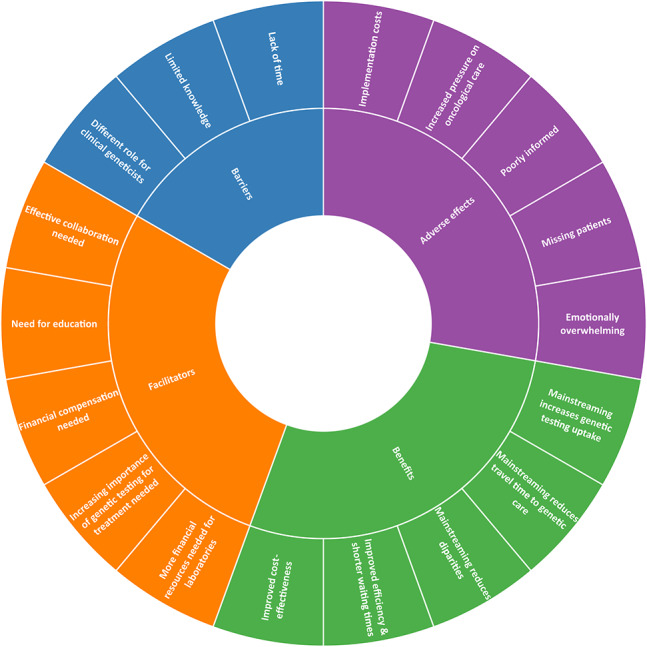
The barriers, facilitators, benefits and adverse effects of mainstreaming based on participants’ perspectives

Among the 18 subthemes, we identified three barriers and five facilitators to the implementation of mainstreaming for medical oncologists and nurse practitioners. We also identified five benefits and five adverse effects of mainstreaming (Fig. 2).

## Discussion

This study identified several potential barriers and facilitators to the implementation of mainstreaming in breast cancer care by medical oncologists and nurse practitioners, as reported by participating relevant stakeholders.

The barriers limited knowledge and lack of time were both also identified in other studies as reasons for medical oncologists being less interested in mainstreaming [17–19]. Enhancing HCPs’ knowledge of genetic testing and the consequences of genetic testing through education has been shown to be effective in improving the confidence and competence of medical oncologists when discussing genetic testing [20]. Regarding the concerns about limited knowledge, those medical oncologists already involved in mainstreaming did not perceive their level of expertise as a barrier. The same applied to concerns about lack of time: those participants already involved found the extra time investment needed for discussing genetic testing with the patient to be acceptable. This is in line with an earlier study in the UK, in which medical oncologists rarely cited their workload as a barrier and expected the incorporation of mainstreaming in oncology would let them provide streamlined care to breast cancer patients [13]. In an earlier Dutch study, some surgical oncologists and nurse practitioners participating in a mainstreaming training programme thought before the training course that pre-test counselling was too time-consuming. After completing the training, less than half the HCPs still found it to be time-consuming [21]. Other studies examining HCPs’ experiences with mainstreaming in ovarian cancer have found that the time investment required for discussing and ordering genetic testing is deemed to be acceptable. These findings suggest that, despite initial perceptions of mainstreaming as time-consuming, the time commitment is ultimately regarded as acceptable by HCPs [22–24]. To minimise the extra time investment for medical oncologists even further, one recommendation could be providing education about discussing genetic testing efficiently; that was found to be a facilitating factor in this study.

Effective collaboration between hospital departments through well-defined agreements, often mentioned by participants, is required for the further implementation of mainstreaming. Cross-departmental collaboration and clear communication pathways have also been emphasised in other studies as essential for the smooth implementation of genetic services in breast cancer care [18]. If mainstreaming is to be implemented successfully, it is essential that all relevant stakeholders are actively involved in the process.

Medical oncologists highlighted the need for financial compensation. Two distinct aspects of financial compensation were mentioned: ensuring that the costs of genetic counselling and testing are covered, preventing financial strain on the oncology department, and compensation for the oncologists themselves to account for the additional workload associated with discussing genetic testing. An earlier study revealed that HCPs cited the lack of reimbursement for extra tasks as a reason for not having a positive attitude to mainstreaming prior to participating in it. Interestingly, six months after completing the mainstreaming training module, HCPs no longer listed this as a barrier [21]. When looking at the implementation costs, the potential costs associated with the logistical expenses involved in implementing mainstreaming (such as educating HCPs and developing protocols) must also be taken into account. However, the majority of participants expected mainstreaming to be cost-effective due to the reduction in consultations with clinical geneticists and the long-term prevention of cancers.

The potential benefits of mainstreaming identified in this study (such as a faster, more efficient and more accessible care pathway, and an increase in genetic testing uptake) are supported by evidence from breast cancer mainstreaming pilot programmes demonstrating similar outcomes [4, 7–9]. The same effects were reported in pancreatic cancer mainstreaming programmes [25]. That the majority of participants expect SES to influence the access to genetic testing and that mainstreaming could help reduce disparities in access is an encouraging finding. These findings are in line with previous findings that mainstreaming has the potential to democratise access by integrating genetic testing into routine care pathways, thereby bypassing the traditionally limited access to specialist genetics services [4]. However, disparities in genetic testing still persist. In addition to introducing mainstreaming, increasing HCPs’ awareness of the current inequitable access to genetic care and further education on recognising patients with low SES are essential in order to reduce these disparities.

Additionally, some potential adverse effects of mainstreaming were mentioned. Some participants noted that having a medical oncologist discuss genetic testing with a patient could be overwhelming, as patients are already required to process substantial amounts of information and cope with emotionally distressing news early in the care pathway. This statement is in line with the views of medical oncologists, who said that patients with metastatic breast cancer are often overlooked due to genetic testing being postponed (and thereby forgotten) as their care pathway is already deemed to be highly overwhelming. Another study found similar concerns, noting that introducing genetic testing at the time of breast cancer diagnosis could lead to emotional and informational overload, with patients struggling to process both cancer and genetic information simultaneously [26]. In a more recent study, however, mainstreaming was demonstrated (despite some differences in care) to provide a solid basis for informed decision-making without causing unacceptable levels of distress or decision regret [21]. Another study found that all ovarian cancer patients who were offered genetic testing during their initial oncology consultation found the timing to be acceptable [27]. It is therefore essential to involve medical oncologists in genetic testing and to engage actively with patients with metastatic breast cancer about their needs and preferences for genetic testing. Clinical geneticists highlighted the risk of missing patients with pathogenic variants in genes associated with rare cancer predisposition syndromes if non-genetic HCPs are responsible for determining eligibility for genetic testing. To mitigate this risk, it is crucial that non-genetic HCPs should be educated about the eligibility criteria to ensure that no patients are overlooked. Additionally, effective collaboration between oncology and genetics departments, identified as a facilitator, could play a key role in mitigating the risk of patients being missed.

A strong point of the study is the inclusion of HCPs from multiple healthcare regions, resulting in a diverse interview cohort, broadly reflecting current care practices. A limitation is that the study did not capture the patients’ perspective. Research exploring the patients’ perspective is therefore needed in future, particularly looking at the needs and preferences of women with stage IV breast cancer. We also only interviewed clinical geneticists who had either been involved in mainstreaming for an extended period or had recently become involved with mainstreaming. We have not yet interviewed clinical geneticists who had no experience with the mainstreaming approach. Another limitation of this study is the potential for self-reporting bias, as responses may have been influenced by social desirability, potentially affecting accuracy and leading participants to provide answers that differ from their true beliefs or experiences. Additionally, self-selection bias may have played a role, as HCPs with a positive attitude toward mainstreaming might have been more inclined to participate in interviews on the topic.

In conclusion, this study highlights key barriers and facilitators in the implementation of mainstreaming in breast cancer care by medical oncologists and oncological nurse practitioners. Lack of time and limited knowledge of genetics were identified as the most important barriers, while effective cross-departmental collaboration and targeted education emerged as crucial facilitators. The potential benefits of mainstreaming, including faster and more efficient processes and increased genetic testing uptake, suggest that integrating it could lead to improved patient outcomes. Potential adverse effects were mentioned, such as the risk that discussing genetic testing with a medical oncologist could be emotionally overwhelming for patients. Although opinions were divided on the influence of socioeconomic status, most participants believed that mainstreaming could help reduce disparities in access to genetic services. These findings point to several context-specific strategies that may help to overcome the reported barriers and support the involvement of medical oncologists in mainstreaming. Education aimed at enhancing oncologists’ ability to efficiently discuss germline genetic testing could help address existing knowledge gaps and time constraints. In addition, well-organized cross-departmental collaborations between medical oncologists and genetic healthcare professionals may contribute to more streamlined pathways. Further research is needed to evaluate how these strategies can be most effectively implemented in practice and what their impact is on patient experience and equity of access.

## Supplementary Information

Below is the link to the electronic supplementary material.

Supplementary Material 1

Supplementary Material 2

## Data Availability

The datasets generated and/or analysed during the current study are not publicly available due to privacy reasons but are available from the corresponding author on reasonable request.
