# Correction: Barriers and facilitators for medical oncologists in the further implementation of mainstream genetic testing in breast cancer care in the Netherlands

**DOI:** 10.1007/s10689-025-00510-7

**Published:** 2025-11-18

**Authors:** Chiem L de Jong, Gina Schijven, Ellen G Engelhardt, Agnes Jager, Margreet G. E. M. Ausems

**Affiliations:** 1 Division Laboratories, Pharmacy and Biomedical Genetics, Department of Genetics, University Medical Center Utrecht, Heidelberglaan 100, 3584 CX Utrecht, The Netherlands; 2 Division of Psychosocial Research and Epidemiology, The Netherlands Cancer Institute, Plesmanlaan 121, 1066 CX Amsterdam, The Netherlands; 3 Department of Medical Oncology, Erasmus University MC Cancer Institute, Dr. Molewaterplein 40, PO Box 2040, 3000 CA Rotterdam, The Netherlands

**Correction to: Familial Cancer (2025) 24:75** 10.1007/s10689-025-00500-9

In this article, the wrong figure appeared in Fig. 2. The incorrect and correct versions of Fig. 2 are provided below:

Incorrect version of Fig. 2:


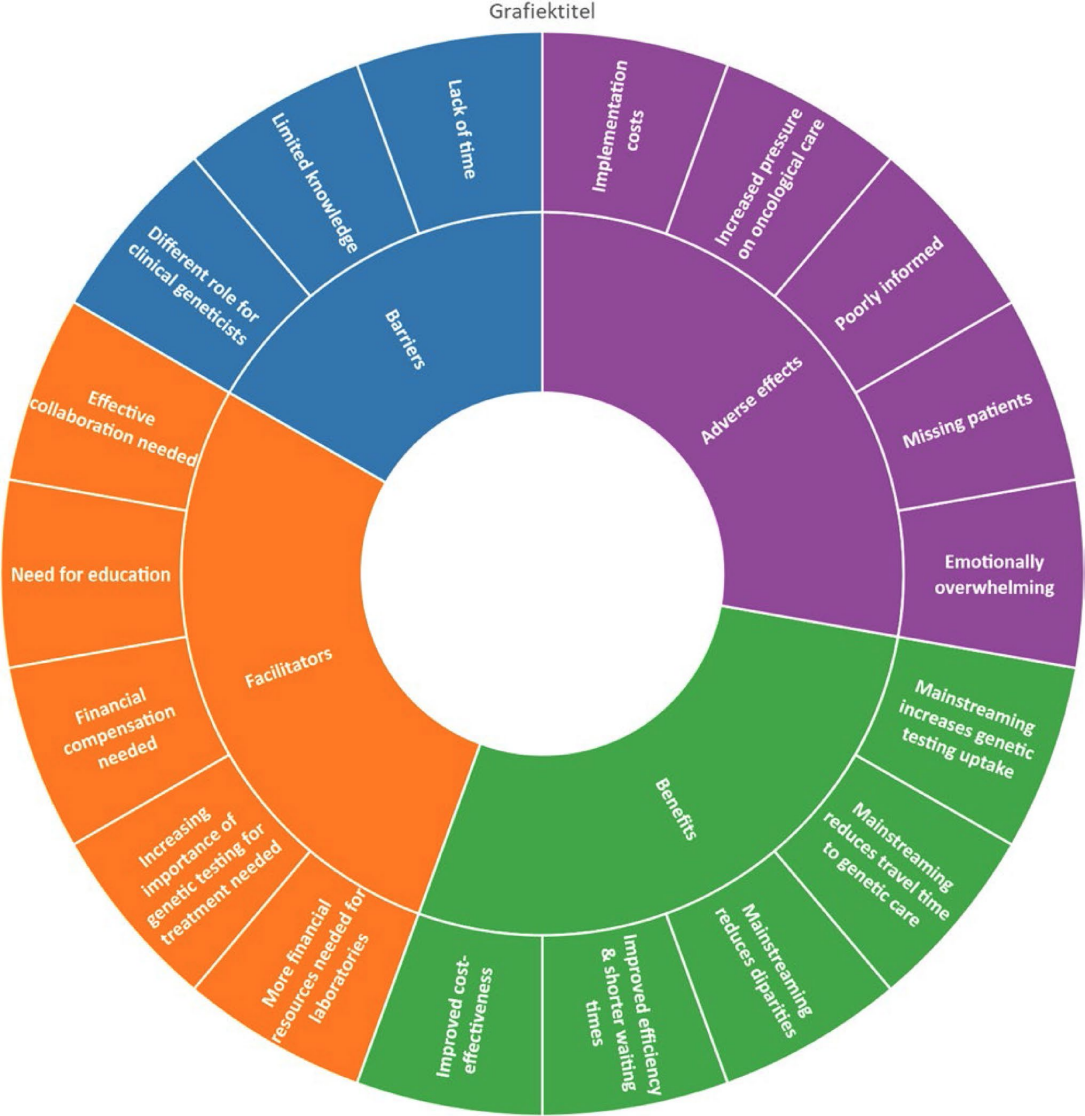


Correct Version of Fig. 2:

**Fig. 2:**
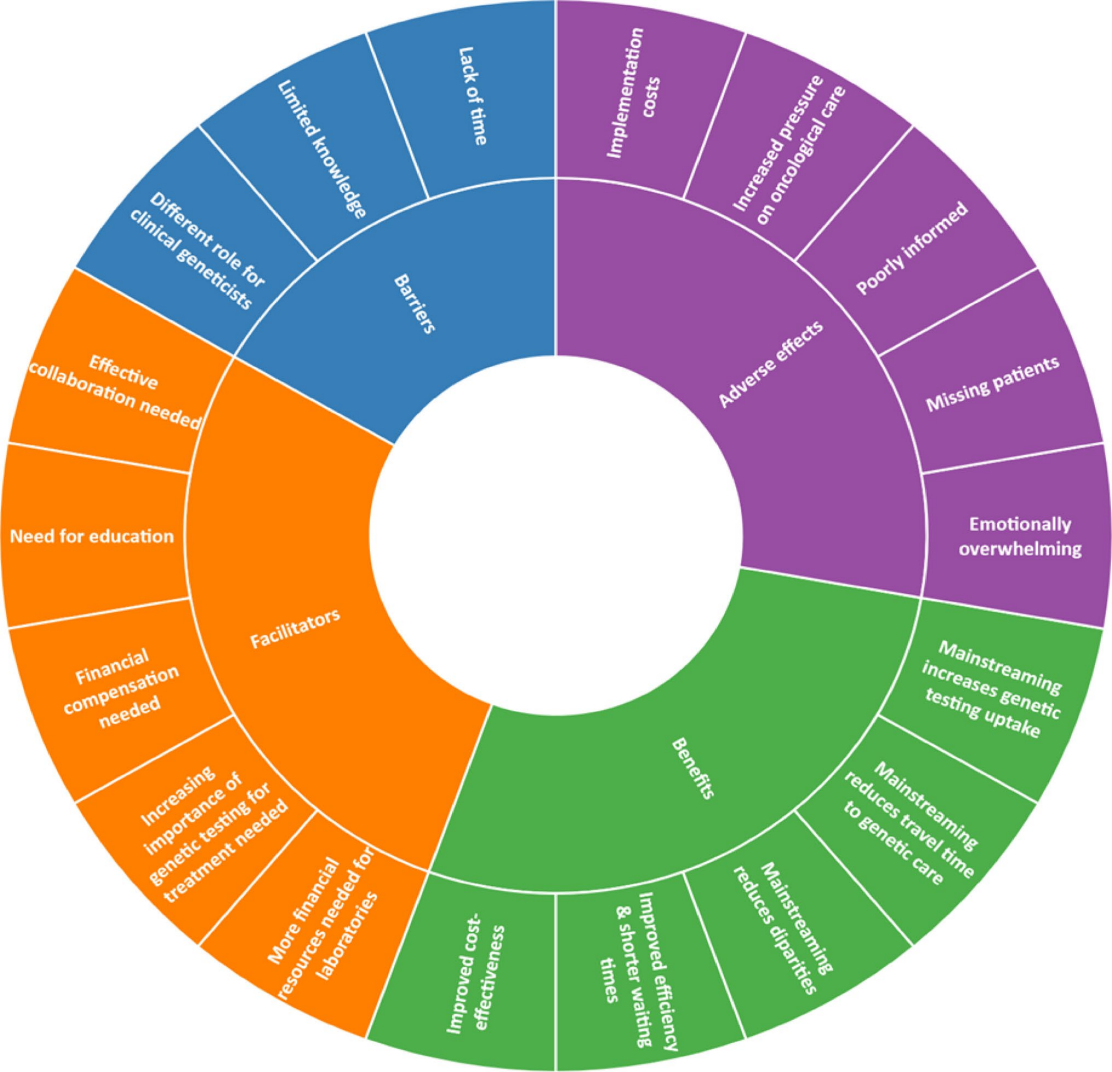
The barriers, facilitators, benefits and adverse effects of mainstreaming based on participants’ perspectives

