# Supplementary material for: Barriers and facilitators for medical oncologists in the further implementation of mainstream genetic testing in breast cancer care in the Netherlands
Source: Fam Cancer. 2025 Oct 14;24(4):75. doi: 10.1007/s10689-025-00500-9 (PMC12521265; doi:10.1007/s10689-025-00500-9)
Supplement: Supplementary file 1 — Supplementary Material 1 [file 10689_2025_500_MOESM1_ESM.docx]

**Part 1: General**

1. **Demographic questions:**
2. Which organisation do you work for?
3. What is your position within that organisation?
   1. What is your role in breast cancer care within your organisation?
4. How long have you been working as a (…) in breast cancer care?

**Part 2: Organisation**

1. **Organisation of genetic testing**
2. How is genetic testing for breast cancer organised in your hospital?
   1. What do you think of this way of organising genetic care?
3. Is a clinical geneticist always present at the multidisciplinary team meeting?
4. Mainstreaming involves informing patients about genetic testing and initiating this testing by non-genetic healthcare professionals. Are you familiar with this approach?
5. Do you ever inform patients about germline genetic testing for hereditary breast cancer and initiate this testing yourself?
   1. **If yes:**
      1. What are your experiences with this process?
         1. How much time do you typically spend per patient on this?
            1. Do you personally decide to spend this amount of time?
            2. **If yes:** Why do you choose to spend this amount of time?
            3. **If no:** Is this a hospital-wide agreement?
         2. Do you feel this is long enough to discuss genetic testing with patients?
            1. Why or why not?
            2. **If no:**

How long would you ideally like to spend on this?

- - 1. Which patients do you initiate genetic testing for?
       1. Based on patient characteristics
       2. Based on family history
       3. A combination of both
       4. Based on therapeutic indications
    2. Were there any challenges or concerns when you first began informing patients about genetic testing (e.g. lack of time, experience, knowledge, reimbursement or perceived responsibility)?
    3. Would you recommend that other non-genetic healthcare professionals should initiate genetic testing?
       1. **If yes:**
          1. Do you have any advice for other non-genetic healthcare professionals on initiating genetic testing?
       2. **If no:**
          1. Why not?
  1. **If no:**
     1. Who is responsible for this in your organisation?
     2. Do you refer patients to a clinical geneticist for genetic testing?
     3. Would you be open to discussing and initiating genetic testing with patients yourself?
        1. **If yes:**
           1. Why are you open to this?
           2. Do you foresee any challenges or difficulties in taking on this responsibility (e.g. lack of time, experience, knowledge, reimbursement or perceived responsibility)?
        2. **If no:**
           1. Why are you not open to this (e.g. lack of time, experience, knowledge, reimbursement or perceived responsibility)?

1. **Do the patients actually get genetic testing?**
   1. **If the respondent discusses and requests genetic testing:**
      1. Do all patients who are eligible for genetic testing according to breast cancer guidelines actually get tested, or have you come across patients for whom genetic testing has been unjustifiably omitted?

Could you describe which types of eligible patients are most often missed? In terms of:

- - - - 1. Socioeconomic status
        2. Health literacy
        3. Cultural background
        4. Ethnicity
  1. **If the respondent refers patients for genetic testing:**
     1. Do all patients who are eligible for genetic testing according to breast cancer guidelines get referred to a clinical geneticist, or have you come across patients for whom this has been unjustifiably omitted?

Could you describe which types of eligible patients are most often missed? In terms of:

1. Socioeconomic status
2. Health literacy
3. Cultural background
4. Ethnicity
5. Do you know if all patients referred to the clinical geneticist actually get germline genetic testing?
6. **Acceptance by healthcare professionals**
   1. What do you think are the barriers for oncologists in implementing mainstreaming?
      1. Can you explain why?
   2. What do you think are facilitators for oncologists in implementing mainstreaming?
      1. Can you explain why?
   3. What do you think would need to happen before mainstreaming can be implemented across the Netherlands?
      1. Why do you think that?
   4. What is your opinion on training nurse practitioners to inform patients about genetic testing and initiate this process?
      1. Why do you think that?
7. **Education/training about discussing genetic testing**
   1. Do you feel competent to inform patients about germline genetic testing and request genetic testing? / Would you feel competent to inform patients about germline genetic testing and request genetic testing?
   2. Is training in germline genetic testing for breast cancer offered within your organisation?
      1. **If yes:**
         1. Do you know what this training entails?
            1. Do you have anything else to say about this training?
         2. Who is the training offered to?
         3. Who provides the training?
         4. How often is the training offered?
         5. Have you been offered this training?
            1. **If yes:**

Did you complete the training?

**If yes:**

What was your experience of this training?

**If no:**

Why not?

- - - - 1. **If no:**

Would you have liked to attend the training if it had been possible?

**If yes:**

What would you like to learn in a training course on mainstream genetic testing?

**If no:**

Why not?

- - 1. **If no:**
       1. Would you have liked to attend the training if it was possible?
          1. **If yes:**

What would you like to learn in a training course on mainstream genetic testing?

How often would you like to follow this training?

- - - - 1. **If no:**

Why not?

**Part 3: Clinical outcomes**

1. **Clinical outcomes of mainstream genetic testing**
   1. How much do you think mainstreaming genetic testing affects the uptake, i.e. the percentage of eligible breast cancer patients who undergo genetic testing?
      1. Why do you think that?
2. **Therapeutic implications**
   1. It is possible that a patient may not formally qualify for genetic testing under current guideline criteria for hereditary breast cancer, yet there could potentially be therapeutic implications for the patient if they are found to carry a BRCA mutation. Have you encountered such cases?
      1. **If yes:**
         1. What are your experiences?
         2. How do you approach such situations?
            1. Why do you approach it this way?
      2. **If no:**
         1. How would you handle such a situation?
            1. Why would you handle it this way?
   2. As the therapeutic indications for genetic testing become increasingly important, demand for germline genetic testing will rise. This could lead to capacity challenges in clinical genetics. Do you believe mainstream genetic testing could help increase the capacity of genetic services?
      1. **If yes:**
         1. How do you think mainstreaming could help?
      2. **If no:**
         1. What barriers do you foresee that might limit capacity expansion?
         2. Why do you see these as barriers?
   3. What do you believe is the impact of mainstreaming genetic testing on the burden of oncological care?
      1. Why do you think that?

**Part 4: Patient-related factors**

1. **Acceptance by patients**

What reasons might patients have for accepting genetic testing and counselling performed by a non-genetic healthcare professional (e.g. a medical oncologist)?

What reasons might patients have for declining genetic testing and counselling performed by a non-genetic healthcare professional (e.g. a medical oncologist)?

Why do you think that?

Could you comment on the turnaround time for your patients who are eligible for genetic testing?

Is there a difference in turnaround time between emergency and non-emergency cases?

What are your thoughts on this?

If there are waiting times for non-emergency cases: what do you think causes these waiting times?

1. **Ethics**
2. To what extent do you think mainstream genetic testing makes genetic care more equitable for breast cancer patients who currently live farther away from university medical centres with clinical genetics departments?
3. Research shows that a patient's socioeconomic status influences referral rates for genetic testing. To what extent do you believe mainstream genetic testing improves accessibility and fairness for breast cancer patients with lower health literacy and/or lower educational levels?

**Deel 5: Economic factors**

1. Are you aware of how germline genetic testing is currently funded?
2. In your view, what are the main costs associated with implementing the mainstreaming approach?
   1. Why do you think that?
3. Diagnostic costs from complex genetic counselling (e.g. clinical genetics consultations) are expected to decrease if mainstream genetic testing is implemented successfully. Can you think of other factors that might influence the cost-effectiveness of mainstream genetic testing?
4. Do you think implementing mainstream genetic testing will have a positive or negative effect on overall cost-effectiveness (including subsequent treatments following diagnostics)?
   1. Why do you think that?
