# Supplementary material for: Barriers and facilitators for medical oncologists in the further implementation of mainstream genetic testing in breast cancer care in the Netherlands
Source: Fam Cancer. 2025 Oct 14;24(4):75. doi: 10.1007/s10689-025-00500-9 (PMC12521265; doi:10.1007/s10689-025-00500-9)
Supplement: Supplementary file 2 — Supplementary Material 2 [file 10689_2025_500_MOESM2_ESM.docx]

**Supplementary Table 1**: Quotes supporting themes.

| **Theme** | **Quote** | **Quote number** |
| --- | --- | --- |
| Lack of time | *“We only have a few minutes — there’s no extra 15 minutes to spare. I have about 40 minutes with a new patient, during which I need to explain their diagnosis, discuss treatment options and now, on top of that, cover genetic testing too?”* | Q1 |
|  | *“You have to provide extra explanation. I think the clinical geneticist has significantly more time during the first consultation than we do to inform the patient about genetic testing.”* | Q2 |
|  | *"I think that you spend about half an hour to an hour altogether on additional explanations and you could see four other patients in that time. So I think that’s quite a challenge."* | Q3 |
|  | *"In a first consultation, I can’t both inform someone about the relative benefits of neoadjuvant chemotherapy or hormonal therapy, for instance, along with all the side effects and the treatment schedule, and then also say, 'Oh yes, by the way, we also need to consider whether there might be a genetic cause’ and what that would mean."* | Q4 |
|  | *"There was a discussion at the time about whether the medical oncologists should take part in mainstream genetic testing or not. Those training e-mails were forwarded as well, but we ultimately decided as a group to leave it with the surgical team for the time being. I think that decision was partly for managing our own workload, because we often already have a lot to discuss when patients come to us, such as chemotherapy. So we decided to leave the DNA testing with them for now."* | Q5 |
| Limited knowledge | *"I think it ultimately comes down to the knowledge aspect: are you sufficiently aware of who is eligible for DNA testing and what you should or shouldn’t discuss with the patient? Are you able to have that conversation effectively?"* | Q6 |
|  | *“Knowledge is of course an important barrier. Common pathogenic variants are not the issue, but I’m less familiar with the newer ones. That’s why I believe it is beneficial to discuss genetic testing with a clinical geneticist.”* | Q7 |
|  | *"I noticed this week, when I was referring a patient, that there are still quite a lot of gaps in my knowledge. I definitely need to learn more about it before I can feel completely confident in that area."* | Q8 |
|  | *"I foresee difficulties with follow-up questions. If I have the genetic testing done, what does it mean if an abnormal result is found? Does it affect my disability insurance or my ability to get a mortgage? What does it mean for my family? Of course, you could say, 'We’ll do the DNA testing first and then see what happens,' but I don’t think that’s the right approach. So, I really feel I need to know more about this before I could handle it properly."* | Q9 |
| Education Needed | *“It’s always helpful to refresh your knowledge about the pathogenic variants. But what I’m particularly interested in is hearing about their experiences: what do you tell a patient about how they should cope with it? There’s the practical aspect of informing your family, of course, and how the patient personally deals with it. It’s really complex because it affects so many areas of your life. It isn’t just, 'Oh, you need to have your breasts removed.' It affects your outlook on the future, your desire to have children... So, I would like to have more guidance on how to support a patient in this. So it’s partly the practical aspects — why we do genetic testing and what it means medically — and also what it means for the patient themselves.”* | Q10 |
|  | *"Some people don’t fully understand how heredity works. So I think training on how to explain this to patients simply and clearly is important."* | Q11 |
|  | *"I think it’s important to focus on how to start such a conversation without scaring people too much. It’s also crucial to explain clearly how it works, as some people find the idea of having a genetic abnormality quite frightening."* | Q12 |
|  | *"It’s also important to receive training about what it means for their lives and the emotional impact it can have. The idea that you could pass something undesirable on to your children is something people can struggle with emotionally."* | Q13 |
|  | *"I think that if we’re really going to start doing this, it would be helpful to begin with a classroom-style training session — getting all the medical oncologists and nurse practitioners together for an afternoon. That way, you can reach the whole group, ask each other questions, and learn from one another. Additionally, I think some on-the-job learning would be valuable, with the option of easily consulting colleagues who have more expertise in this area."* | Q14 |
|  | *"If you just start doing it and do it regularly, you’ll learn as you go."* | Q15 |
| Financial compensation needed | *"If it’s handled by the medical oncologists, it’s important to make sure that they receive appropriate compensation for the work they do."* | Q16 |
|  | *"Ultimately, there are only so many hours in a day. So I think it would only be fair if the time and effort are appropriately compensated, irrespective of whether it’s the medical oncologist or the nurse practitioner taking on that additional work. I believe that would be justified."* | Q17 |
|  | *"It’s an investment, of course. If it’s going to take extra time, I can imagine that medical oncologists would prefer not to take it on unless it’s properly compensated. I think the issue of compensation needs to be considered, as it’s always a good incentive — you simply want to be paid for the work you do."* | Q18 |
| Increasing importance of genetic testing for treatment needed | *"What is also a facilitating factor is that it has treatment consequences. We want to actually use the results, which is why it’s now being done more frequently by medical oncologists. At least, because there is treatment specifically for patients with a pathogenic variant. That’s why it’s becoming more relevant for medical oncologists."* | Q19 |
|  | *"If the results of genetic testing become more important in terms of treatment consequences, that could also be a facilitating factor."* | Q20 |
| Missing patients | *"Well, I don’t get the impression that we’re missing anyone. I think it’s being done quite frequently. There are certainly some patients who don’t want it, but that’s really a minority among breast cancer patients; it’s more common with other types of cancer. I don’t feel that it’s often overlooked or not being done."* | Q21 |
|  | *"I don’t get the impression that we’re missing patients. We’re quite proactive in referring patients for genetic testing. However, the patient does need to give their consent, of course. But genetic testing is almost always offered in principle, as soon as a patient is eligible. After all, why wouldn’t it be?"* | Q22 |
|  | *"Of course, there may be occasions when a patient is missed. However, it’s included in our MDT report, so it’s always discussed in the MDT meeting: is there an indication for genetic testing? And then everyone contributes to that decision. So I expect that very few people are actually missed."* | Q23 |
|  | *"Generally, it’s older patients who get missed. Not patients under 40 — those are almost always considered. I think it’s primarily patients over 50."* | Q24 |
|  | *"Patients who might be missed are, for example, those with triple-negative breast cancer who are a bit older, where the family history suddenly becomes important, but that’s not something you immediately consider. This could happen, for instance, when someone is already undergoing treatment."* | Q25 |
|  | *"For example, patients with triple-negative breast cancer just under 60 years old with a negative family history — those are sometimes overlooked. Or patients who had breast cancer in the past and come back at 70 with breast cancer again. These patients had breast cancer 20 years ago, so it is bilateral breast cancer, which suddenly makes them eligible for genetic testing. That’s where things can sometimes go wrong."* | Q26 |
|  | *"The problem, I think, arises when something changes in the patient’s family over time, and the patient has been known for a longer period. Suddenly, a diagnosis of breast cancer in a sister or aunt emerges, or prostate or pancreatic cancer in another family member, and the patient doesn’t realise they need to report it. Doctors don’t always actively ask if anything has changed in the family. I try to do so, but undoubtedly it will happen to me as well. I think the risk lies less in the primary diagnosis and more in the follow-up, when something changes in the family and no one thinks to consider it."* | Q27 |
|  | *“So much has to be discussed, and it makes you think, ‘Let’s talk about this next time.’ But then the next time, I might forget to bring it up. It is also not always at the onset of the disease. People already have enough to process, so it might also be, ‘I’ll put it aside until it becomes more relevant.’”* | Q28 |
|  | *"I think that if patients are missed, it would be patients with* de novo *metastatic breast cancer who don't come through the surgical route."* | Q29 |
|  | *"The only situation I can imagine is if a patient turns out to have metastatic breast cancer but they aren’t discussed in an MDT meeting because the medical oncologist has already started the treatment independently."* | Q30 |
|  | *“I’m afraid that you might miss potential rare pathogenic variants. We need to be cautious about missing the rarer abnormalities that aren't so obvious and aren't included in the standard panel.”* | Q31 |
|  | *“It’s possible that you might miss potential clues in the family that point to a hereditary cause. Clinical geneticists also look more broadly at the partner’s family, of course, especially where children are concerned. So yes, that fine-tuning, which is important, might get lost in the process.”* | Q32 |
| Mainstreaming increases genetic testing uptake | *"I definitely don’t think we’re doing less genetic testing. When you take on a more active role, I think you forget about it less often. Personally, I feel that it makes us more proactive in thinking about genetics, because it then becomes partly our responsibility."* | Q33 |
|  | *"Well, my gut feeling would be that we find more patients than when we refer for genetic testing, because when mainstream genetic testing is implemented, it gets much easier to request it."* | Q34 |
|  | *"The uptake increases. I’m pretty certain of that, in fact; it’s simply the case."* | Q35 |
|  | *"I think that far fewer patients are missed as a result because it’s now included in the workflow of the care pathway. And, well, if you refer a patient to a clinical geneticist and there’s a waiting list, I can also imagine that the patient might eventually say, 'Forget it.'"* | Q36 |
|  | *"I think the uptake is higher because you already have a treatment relationship with the patient, which makes it easier to emphasise how important the testing is. Plus, the patient doesn’t need to go to another hospital for an appointment. I think it’s just much more practical. And in the case of mainstreaming, the blood sample is also taken alongside the work that already needs to be done."* | Q37 |
|  | *“You get the diagnosis of breast cancer, and then you enter a madhouse where patients have no idea what’s happening to them. They get a zillion appointments and have to make so many decisions... If you can avoid having to see one doctor, especially one who’s going to explain a complicated story, that’s actually quite a relief.”* | Q38 |
|  | *"When the patient is referred for genetic testing, it means an additional visit to another specialist for them. I wouldn't say that it’s hugely discouraging, but it does seem that it weighed more heavily for people than it does now when a simple blood sample can be taken."* | Q39 |
|  | *"Well, for example, today I had a patient who still found it quite a hurdle to see the clinical geneticist. And because I’m a familiar face, it can be helpful if I can just request the genetic testing myself."* | Q40 |
|  | *"A patient with breast cancer already has so many interactions with healthcare providers in the first few weeks – I think it’s already too much. So if a clinical geneticist is added to the list, it can become overwhelming for the patient."* | Q41 |
|  | *"For the patient, referral to a clinical geneticist means that an additional doctor is brought into the picture. It might be more convenient for the patient if the doctor they are already seeing takes care of all of that."* | Q42 |
| Mainstreaming is emotionally overwhelming | *"People are different. Some patients want to know everything from the start, whereas others find it all overwhelming. They want to take things step by step. And when this comes up as well, it feels like yet one more thing for them to deal with."* | Q43 |
|  | *"I think it’s sometimes also the stage of the care pathway where patients reckon that they can't handle thinking about that right now. That can be a factor."* | Q44 |
|  | *"Sometimes I feel like it's an information overload in a very short time. The patient gets so much information in the first month that they can barely keep up. If you're young, you might still have fertility concerns, so you go to the gynaecologist. You see the surgeon, the radiologist, the radiation oncologist, the plastic surgeon, and the medical oncologist. Often, each specialist requires two consultations. And that’s without even mentioning the additional tests that need to be done. It's just a lot for patients to process in such a short period."* | Q45 |
|  | *"You're on a huge rollercoaster, of course: 'I have breast cancer, I need treatment, what does this all mean?' And then genetics is also added to the mix. I can imagine that it can feel overwhelming for the patient. But in practice, it’s usually not as much of an issue as you might think."* | Q46 |
| Poorly informed | *"Patients might say afterwards, 'I wish I’d never known.' That could happen — there may be more people who feel they weren't adequately informed about the consequences and then regret having had the test done."* | Q47 |
|  | *"One downside could be that you don’t get enough information about the consequences of genetic testing before you decide to have it done.”* | Q48 |
|  | *"Of course, there are always patients who would benefit from an extra appointment with the clinical geneticist and who need a specialist’s perspective during the intake. Those patients will remain."* | Q49 |
| Mainstreaming reduces disparities | *"I think it mainly matters for patients who don’t formally meet the referral criteria, because highly educated patients may be more likely to bring up family cancer history during a consultation."* | Q50 |
|  | *"In general, I think highly educated patients are more alert and will bring it up themselves earlier, saying things like, 'My sister... shouldn’t she be tested too?' I don’t formally refer borderline cases, but I’ll still consult a clinical geneticist. The clinical geneticist is usually flexible and will often agree to genetic testing being requested. I think patients with lower socioeconomic status are less likely to ask that question and don’t push as much, which leads you to think they don’t meet the criteria, so you won’t request genetic testing."* | Q51 |
|  | *"One possibility is that some people are unaware of their background, or foreign patients who have no contact with their family, which results in missing information. If you don't have enough reason to request genetic testing, then you won’t ask for it."* | Q52 |
|  | *"I think people with limited health literacy may not see the value of an extra consultation with a clinical geneticist. You can’t explain to them what the clinical geneticist does and why it’s important to travel for an hour by train and bus to a university hospital—they really won’t do it."* | Q53 |
|  | *"I think that language barriers or low health literacy are definitely issues because it's something new. And DNA is also really complicated – the word 'DNA' alone already puts half the Netherlands off – and even healthcare providers don’t always fully understand how it works. So, if you as a healthcare provider can’t explain it properly, it doesn’t come across clearly to the patient either."* | Q54 |
|  | *“I wonder if we are perhaps so biased in the consultation room that we ask those patients fewer questions.”* | Q55 |
|  | *"I think it’s really helpful for someone with lower health literacy that we can discuss and request genetic testing, because they then don’t have to deal with more people having an opinion or getting involved. I can imagine that it’s reassuring to be informed about it by a familiar point of contact."* | Q56 |
|  | *"I think mainstreaming promotes equality by offering genetic testing in an accessible way to everyone who meets the criteria. Otherwise, you end up with a selection of well-informed, highly educated patients who are willing to travel for it."* | Q57 |
|  | *"For example, I have an academic study going on here, and sometimes patients are willing to talk, but after the discussion, they say that they think the hospital is too big and too far away. These patients live quite close by, I’d have said, but the distance is too much for them and they prefer to stay in their local hospital. For people who have limited mobility or those who don’t have much money or are in debt restructuring, I reckon it saves them time and energy if we can take their blood at a nearby hospital. It really reduces costs for them."* | Q58 |
|  | *"I do think that mainstreaming improves accessibility to genetic care for patients with a lower socioeconomic status. If a patient can’t afford the bus or train fare, they might not go. Mainstreaming helps address this issue."* | Q59 |
| Mainstreaming reduces travel time to genetic care | *"Some women who need to see the clinical geneticist simply don’t have transportation, so it’s really helpful that the clinical geneticist sometimes has consultation hours here, as it removes that barrier."* | Q60 |
|  | *"I do think that if there’s no mainstreaming and someone has to drive an hour just to get a blood sample taken, it will result in some patients dropping out."* | Q61 |
|  | *"It's hard to imagine sometimes, but cities can be big and intimidating places if you have to figure out where to go and search for a parking spot…"* | Q62 |
|  | *"Well, we do a lot of video counselling, which makes it much easier to counsel people. And a lot people say the same thing. I have a lot of patients who live a long way from the hospital, and they’re happy not to have to travel an hour each way and be able to do it via video call instead."* | Q63 |
|  | *"To be honest, I think that's a bit of nonsense in the Netherlands. We have a hundred hospitals and several academic centres. You don’t really have to travel very far. You can also have a video consultation, so I can't imagine that travel distance is a significant barrier for patients in the Netherlands."* | Q64 |
|  | *"In oncology, patients with lower socioeconomic status have less access to specialist care. They have fewer opportunities to travel, they don’t want to, they can’t, or it’s too expensive. That’s a fairly large group of patients for whom this is the case, and it applies to genetic care as well, which is centralised. So doing this lets us bring care closer to the patients, making it accessible to everyone, as it should be."* | Q65 |
|  | *"If the patient has to spend two hours on the bus and doesn't have the money for it, and then decides to skip the appointment, it certainly helps if they can simply have the test done at their own hospital."* | Q66 |
| Effective collaboration needed | *“What is important is that you agree within your hospital on how to implement mainstream genetic testing in your care pathway. We made agreements about that: who takes the patient history, who initially requests the clinical genetic testing. When we implemented mainstream genetic testing, we involved all the relevant parties in that process.”* | Q67 |
|  | *"I think it needs to be very clear who requests what, and when."* | Q68 |
|  | *"I think it's essential to agree within the care pathway who takes the lead, so that it doesn’t get forgotten or done twice by accident."* | Q69 |
|  | *"You really need to collaborate properly with your colleagues. And then it can be truly fantastic. I definitely see the added value for the patient, but you need to have very direct lines of communication within your region; that's something I'm currently working on by positioning people in such a way that there are good contacts and by having people who are truly dedicated to genetics. You really need to have a shared goal for that."* | Q70 |
| Improved efficiency & shorter waiting times | *“I think the biggest advantage is that I don’t have to wait for the clinical geneticist. I don’t need to check if the appointment with the clinical geneticist has actually been scheduled, or call them to ask if it was clear that this was an urgent request.”* | Q72 |
|  | *"Mainstreaming gives clear results within ten days, so I think this is a very efficient and quick approach."* | Q73 |
|  | *"The big advantage, in my opinion, is that you're not waiting for the clinical geneticist: you don't have to check if that appointment has actually been scheduled, and you don't have to follow up on it. All those things — if we can keep them in our own hands, it's much easier."* | Q74 |
|  | *"It just goes faster, because there is essentially no delay in initiating the genetic testing anymore."* | Q75 |
|  | *"I don't understand why hospitals would be against mainstreaming. We get quick results, and if we need a faster result because of consequences for the type of surgery, we can prioritise it. If needed, you can have the results within two weeks. I think that's excellent service."* | Q76 |
|  | *"Since the project started, there are no waiting times because patients can simply have a blood sample taken and the results are available within one or two weeks. That's just incredibly fast."* | Q77 |
|  | *"Mainstreaming naturally takes a lot of urgent cases off the hands of clinical geneticists, allowing them to address their waiting lists more effectively."* | Q78 |
|  | *"Some urgent requests now go through mainstreaming instead of us. This primarily affects the regular waiting times — patients who would otherwise wait three months for a standard appointment. With mainstreaming, fewer people are on the waiting list, because part of the workload is redirected."* | Q79 |
|  | *“There will be more space to see patients who are particularly complex and not picked up by standard diagnostics. It allows time for other tasks, such as MDTs. It's perfectly fine if the routine work is handled by others.”* | Q80 |
|  | *"Clinical geneticists generally have waiting lists and mainstreaming is, of course, a much more efficient procedure. If no abnormality is found, the clinical geneticist doesn’t need to take further action. However, if a pathogenic variant is identified, the clinical geneticist can provide further counselling."* | Q81 |
| Improved cost-effectiveness | *"Ultimately, from a broader healthcare perspective, it will be cost-effective. You’re removing a step — referrals to clinical genetics that aren’t necessary and that are expensive. You’ll identify more people who meet the criteria, with a high yield of mutations. These individuals can be helped personally, as can others within their families, which is good in terms of prevention. So I think it will have a positive impact in the end."* | Q82 |
|  | *"The fact that a clinical genetics consultation can be omitted for some patients will impact costs positively."* | Q83 |
|  | *"My gut feeling suggests it should already be cheaper, as the majority of patients don’t have a mutation, and those patients won’t require a consultation with the clinical geneticist."* | Q84 |
|  | *"It will be cost-effective because you avoid all those initial consultations for patients with a negative test. This makes the process much more efficient."* | Q85 |
|  | *"If we have more samples, we can pool them more efficiently. A kind of bottleneck for us is ensuring that these chips on a sequencer are fully loaded. These chips are very expensive, costing around €25,000 each. So, I don’t want only half a load on a chip — it’s simply a waste of money. The more samples we have, the faster we can fill the chip, which is definitely an advantage."* | Q86 |
|  | *"What we reimburse for medical oncologists or surgeons is much cheaper than what we pay clinical geneticists. We negotiate much more competitive rates with medical oncologists and surgeons."* | Q87 |
| Implementation costs | *"Training the staff will also cost. For instance, an educational video was created at the time, which often involves expenses. Moreover, these videos need regular updates to stay up to date."* | Q88 |
|  | *"Developing protocols and creating new forms, those kinds of tasks certainly cost, particularly the logistical aspects."* | Q89 |
|  | *"If you receive unjustified requests because medical oncologists are looking for a treatment method, then you will get relatively more requests for genetic testing than would actually be necessary."* | Q90 |
|  | *"It is possible that you incur more costs due to unjustified requests because medical oncologists are more accustomed to simply ticking a box for potential tests."* | Q91 |
|  | *"I think that if you request more genetic testing overall, it will result in higher costs macroeconomically."* | Q92 |
| Increased pressure on oncological care | *“We talked about it yesterday: how do we stay energised for this, because it’s obviously not working this way. You end up running late with your consultations, and then the next doctor can’t use the room. It all has to stay manageable and enjoyable; otherwise colleagues will start leaving. So time really is a big issue.”* | Q93 |
|  | *"The burden on oncological care does increase because you need to have more discussions with patients and get informed consent, as you are doing further diagnostics that can have significant consequences for both the patient and their family."* | Q94 |
|  | *"If you move a task, it will create additional work elsewhere. And I think you need to consider that space needs to be created for it, and it should also be seen as an extra task for someone else. Because it's easy to shift a task, but ultimately you're just moving the problem, which is that we may not have enough time."* | Q95 |
|  | *“It will obviously take extra time for discussions and requests. I think it will probably cost five to ten minutes or so. So it could certainly become a burden on oncological care.”* | Q96 |
| Different role for clinical geneticist | *"The thing is, you still maintain the control advice. So, you end up doing a lot of extra work for free, like what they do in another academic hospital, where they for example perform a CanRisk during an MDT meeting."* | Q97 |
| More financial resources needed for laboratories | *"We’re consistently exceeding our production ceiling, and every year there is the challenge of determining when the tipping point will occur. Usually, from November onwards, we are essentially conducting the research for free. This limits our potential for growth, as we cannot simply increase our output without additional funding."* | Q98 |
|  | *"The growth in capacity is entirely dependent on funding. In terms of space and equipment, I don't foresee any issues."* | Q99 |
|  | *"Breast cancer diagnostics in the lab are fully automated. We generate results automatically based on the data and manual adjustment is only required when we find a variant we're unfamiliar with. We've eliminated as much manual work as possible, which has created a lot of room for growth. Theoretically, there's quite a bit of scope for expansion. Nowadays, the machines are so large, making scalability much easier. So, the primary limiting factor is financial.* | Q100 |
|  |  |  |
|  |  |  |
|  |  |  |
